# Activated E2F activity induces cell death in papillary thyroid carcinoma K1 cells with enhanced Wnt signaling

**DOI:** 10.1371/journal.pone.0178908

**Published:** 2017-06-01

**Authors:** Dong Yang, Chuanjiang Wang, Yingwei Luo, Xuan Li, Qingbin Song, Jian Zhang, Shijie Xin

**Affiliations:** Department of Thyroid Surgery, the First Hospital of China Medical University, Shenyang, Liaoning, China; University of Alabama at Birmingham, UNITED STATES

## Abstract

Disruption of Wnt signaling often happens in tumorigenesis, but whether Wnt signaling affects the early stages of thyroid tumor, such as papillary thyroid carcinoma, is still a question, especially in the papillary thyroid carcinoma without genomic RET/PTC mutation. In this study, we demonstrated the important function of Wnt signaling in papillary thyroid carcinoma K1 cells, which have no RET/PTC mutation. We found that K1 cells have enhanced Wnt signaling in comparison to normal thyroid cells. We further demonstrated that K1 cells require the enhanced Wnt signaling for growth and survival. Interestingly, we identified that enhancing E2F activity by either knockdown of Rb or overexpression of Cyclin D1 induces cell death in K1 cells. And we further revealed that the cell death is caused by enhanced oxidative stress. Our studies present a novel cell model to support the key roles of Wnt signaling in early stage of thyroid tumor, and also provide an alternative way to limit thyroid cancer.

## Introduction

Thyroid cancer is the most common malign endocrine neoplasm originating from follicular or parafollicular thyroid cells. Follicular thyroid cells derived from histological subtypes are differentiated thyroid carcinoma including follicular thyroid carcinoma (FTC) and papillary thyroid carcinoma (PTC), and poorly differentiated thyroid carcinoma and anaplastic thyroid carcinoma (ATC). Among it, PTC is the most frequent type of thyroid cancer constituting 75–85% of all cases. PTCs often have genetic alterations such as point mutations of BRAF (B-Raf proto-oncogene) and RAS genes, and RET/PTC rearrangements [1]. However, the molecular mechanism for thyroid carcinogenesis is poorly understood.

The Wnt/β-catenin signaling pathway regulates stem cell pluripotency and cell fate decisions during development. Disruption of this pathway has been suggested in tumorigenesis. In the absence of Wnt signaling, β-catenin is phosphorylated and interacted with glycogen synthase kinase-3β (GSK-3β), Axin, and adenomatous polyposis coli (APC) leading to subsequent proteasomal degradation. Activation of Wnt signaling leads to the increased level of free β-catenin. The free β-catenin translocates to the nucleus with T-cell factor (Tcf)/lymphoid enhancer factor (LEF), and activates transcription of target genes in cell growth control. Activation of Wnt signaling has been reported in colorectal cancer, hepatocellular carcinoma, and endometrial carcinoma [2,3]. Although it is well accepted that altered Wnt signaling is a late event in thyroid cell transformation, as mutation in β-catenin was often found in later poorly differentiated and ATCs, recent studies suggested Wnt signaling is also altered in PTC cells with RET/PTC mutations [4,5,6]. It indicates the importance of the Wnt/β-catenin pathway in the initiation of thyroid cancer. But the role of Wnt signaling in other PTC cells is largely unknown.

In this study, we investigated the functional roles of Wnt signaling in K1 cells, which is one of PTC cells without RET/PTC mutations. By directly comparing Wnt signaling activity between normal thyroid cells Nthy-ori 3–1 and K1 cells, we found K1 cells have significantly higher level of Wnt signaling activity. We further found that the enhanced Wnt signaling is required for the growth and survival of K1 cells. More interestingly, we identified cell death effect in K1 cells by enhancing E2F activity using either knockdown expression of Rb (retinoblastoma protein) or overexpression of Cyclin D1. Furthermore, we revealed that the cell death effect is induced by enhanced oxidative stress in cells. These results help to understand the functional roles of Wnt signaling in PTC cells, and provide an alternative way to kill PTC cells.

## Materials and methods

### Cell culture

Nthy-ori 3–1 and K1 cells were obtained from the American Type Culture Collection (Rockville, MD), and cultured in RPMI medium supplemented with 10% fetal bovine serum (FBS), 50 IU penicillin/streptomycin, and 2 mmol/l L-glutamine from Invitrogen (Carlsbad, CA). All the cells were maintained in a humidified atmosphere with 5% CO_2_ at 37°C.

### Plasmid and lentiviral preparation and transduction

The DN-TCF4 was amplified by the primers DN-TCF4 forward: 5’- GGCGGATCCATGAGCTCCGAAAACTCCTCGGCAG -3’ and DN-TCF4 reverse: 5’- GGCGGATCCCTATTCTAAAGACTTGGTGACGAGC-3’. The TCF4 was amplified by the primers forward: 5’-GGCGGATCCATGCCGCAGCTGAACGGCGG-3’ and DN-TCF4 reverse primer. The Cyclin D1 was amplified by the primers Cyclin D1 forward: 5’- GGCGGATCCATGGAACACCAGCTCCTGTGC -3’ and Cyclin D1 reverse: 5’- GGCGGATCCTCAGATGTCCACGTCCCGCACG-3’. The SOD2 was amplified by the primers SOD2 forward: 5’-GGCGAATTCATGTTGAGCCGGGCAGTGTGC-3’ and SOD2 reverse: 5’-GGCGAATTCTTACTTTTTGCAAGCCATGTATC-3’. The PCR fragments were digested and cloned into the lentiviral expression vector pCDH-CMV-EF1-puro from System Biosciences (Mountain View, CA). The pLKO.1 lentiviral RNAi expression system was used to construct lentiviral shRNA. The sequence of shRNA used in this study was described in previous studies [7]. All the constructs were verified by sequencing. Production of lentivirus was performed as described [8].

### Western blot

Cell lysate was prepared in RIPA buffer (50 mM Tris-HCl pH8.0, 150 mM NaCl, 0.1% SDS, and 0.5% Na deoxycholate, 1% NP40) with fresh proteinase inhibitor. The membrane fraction was extracted using Mem-PER Plus Membrane Protein Extraction Kit from ThermoFisher Scientific (Waltham, MA) according to the manufacturer’s manual. The nuclear and cytoplasmic fractions were extracted using NE-PER Nuclear and Cytoplasmic Extraction Reagents from ThermoFisher Scientific (Waltham, MA) according to the manufacturer’s manual. Samples were quantified by Bradford reagent from Sigma (St. Louis, MO) and measured at 595 nm with a microplate reader. Equal amount of protein was loaded. Western detection was carried out using a Li-Cor Odyssey image reader by software Image Studio (Ver. 2.1) from Li-Cor (Lincoln, NE). Antibodies used: β-catenin (D10A8, dilution 1:1000), E-Cadherin (24E10, dilution 1:1000), Histone H3 (D1H2, dilution 1:2000), Cyclin D1(2922, dilution 1:1000), Na,K-ATPase (3010, dilution 1:1000), Phospho-Rb (9307, dilution 1:1000) from Cell Signaling (Danvers, MA), Myc (9E10, dilution 1:1000), β-Actin (AC-15, dilution 1:3000) from Santa Cruz (Dallas, TX), Goat anti-mouse/Rabbit IRDye (dilution 1:10000) from Li-Cor (Lincoln, NE).

### RNA isolation and qRT-PCR

Total RNA was isolated from cells using TRIzol from Invitrogen (Carlsbad, CA) for RT-PCR. Total RNA (2 μg) was reverse-transcribed using M-MLV reverse transcriptase from Promega (Madison, WI) and random primers following manufacturer’s protocol. PCR was performed in triplicate using SYBR green mix from Biotool (Houston,TX), and Real-Time PCR System from Bio-rad (Hercules, CA) under the following conditions: 3 min at 95°C followed by 45 cycles of 95°C for 20 s 60°C for 30 s and 65°C for 1 min. Primers used: MYC forward: 5’-GGCTCCTGGCAAAAGGTCA-3’, MYC reverse: 5’- CTGCGTAGTTGTGCTGATGT-3’; CCND1 forward: 5’-GCTGCGAAGTGGAAACCATC-3’, CCND1 reverse: 5’-CCTCCTTCTGCACACATTTGAA-3’; SOD2 forward: 5’-GGAAGCCATCAAACGTGACTT-3’, SOD2 reverse: 5’-CCCGTTCCTTATTGAAACCAAGC-3’; Catalase forward: 5’-TGTTGCTGGAGAATCGGGTTC-3’, Catalase reverse: 5’-TCCCAGTTACCATCTTCTGTGTA-3’; GAPDH forward: 5’-CTCTGACTTCAACAGCGACAC-3’, GAPDH reverse: 5’-CATACCAGGAAATGAGCTTGACAA -3’. The expression fold changes were analyzed by double delta Ct analysis.

### Transcriptional reporter assay

Cells were treated with lentivirus as described above, and were plated into a 24-well plate, followed by transfection by lipofetamine 2000 from Invitrogen (Carlsbad, CA) according to the manufacturer’s instruction. Luciferase activity was measure using Dual Luciferase Reporter Assay System from Promega (Madison, WI) according to the manufacturer’s instruction. Luciferase activity was read on a BD Monolight 3010 Luminometer. All data points presented are the average measurement of three independent repeats.

### Cell proliferation analysis

Cell proliferation was assessed by cell number counting or MTT assay. For cell number counting, cells were seeded into 6-well plates, and cell number was counted at the indicated day using a hemocytometer after trypan blue staining. For MTT assay, cells were seeded into 96-well plates. After treatments described in result section, culture medium was replaced with fresh medium containing 0.5 mg/ml MTT and incubated for 2 hours at 37°C. After removing the medium, 100 μl of DMSO was added to each well to solubilize the formazan present in viable cells. The plates were analyzed by measuring the optical density at 540 nm.

### FACS analysis of cell death

Cells were seeded into 6-well plates. After indicated treatment, the adherent and detached cells were collected. The cell death was measured by FITC Annexin V Apoptosis Detection Kit from BD Biosciences (San Jose, CA) according the manufacturer’s instructions. The cell mixture was washed twice with cold PBS, and then resuspended at 1×10^6^ cells/mL in binding buffer. Annexin V-FITC and propidium iodide were added to the cell suspension. After gently mixing, the cells were incubated for 15 min at room temperature, and then 400 μl of binding buffer was added to each sample. Quantification of cell death was performed using FACScan from BD Biosciences (San Jose, CA). PI- and/or Annexin V-positive cells were considered as cell death.

### ROS assay

ROS production in cells was measured with Dihydroergotamine (DHE) staining on coverslips. Briefly, cells were plated on coverslips in 6-well plates. After indicated treatment, cells were incubated with medium containing 10 μM of DHE from Sigma (St. Louis, MO) for 30 minutes. The cells were fixed for 30 min in 4% paraformaldehyde, and imaged with a Zeiss fluorescence microscope. The percentage of DHE-positive cells in samples was determined by counting 300–500 cells from 3–5 random fields per coverslip.

### Statistical analysis

The data were collected from at least three independent experiments. Values are presented as mean ± S.D. Statistical significance was assessed by Student’s two-tailed *t*-test and p<0.05 was considered statistically significant.

## Results

### PTC K1 cells have enhanced Wnt signaling

**Fig 1:**
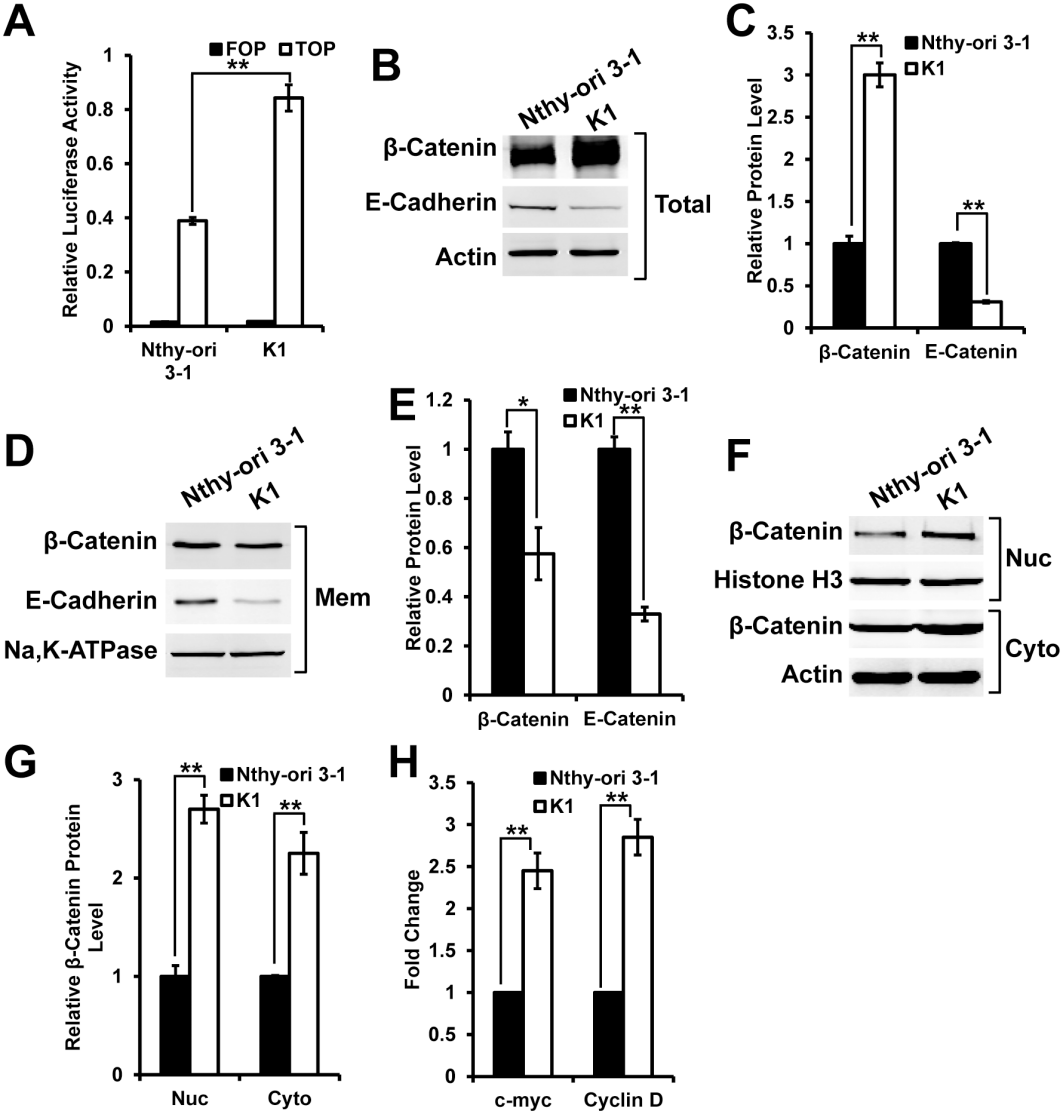
K1 cells have enhanced Wnt signaling. (A) Wnt signaling activity reporter TOP-Flash reporter showed K1 cells have higher Wnt signaling activity than normal thyroid Nthy-ori 3–1 cells. FOP-Flash served as a control; (B) Immuno blot showed increased β-catenin level and decreased E-cadherin level in whole cell extraction from K1 cells. Actin serves as a loading control; (C) Quantification of band intensity in (B); (D) Immuno blot showed decreased β-catenin level and E-cadherin level in membrane extraction from K1 cells. Na,K-ATPase serves as a loading control; (E) Quantification of band intensity in (D) (F) Immuno blot showed increased β-catenin level in both nuclear and cytoplasmic extraction from K1 cells. Actin and Histone H3 serves as loading controls; (G) Quantification of band intensity in (F); (H) mRNA expression of c-Myc and Cyclin D1 relative to the level of GAPDH in Nthy-ori 3–1 and K1 cells. The data were collected from at least three independent experiments. **<0.01.

Accumulated evidences suggest that Wnt signaling might promote the development of PTC. So we tested that whether PTC K1 cells have higher Wnt signaling activity than normal thyroid cells. To measure the activity of Wnt signaling, we used TOP/FOP-Flash reporter system which is a luciferase reporter of β-catenin-mediated transcriptional activation. We respectively transfected the reporters to normal thyroid follicular epithelial cells Nthy-ori 3–1 and K1, and processed the cells for dual luciferase assay. As a control, no significant Wnt activity was detected in FOP-Flash reporter transfected cells (Fig 1A). However, in TOP-Flash reporter transfection set, we found that K1 cells have significantly higher level of Wnt signaling activity than Nthy-ori 3–1 cells (Fig 1A). Considering that most cells respond to Wnt signaling by an increase in the levels of β-catenin, we next compared the level of β-catenin in Nthy-ori 3–1 and K1 cells. The western blot results from whole cell extraction showed that the level of β-catenin in K1 cells is significantly increased, and is about three folds to the level in Nthy-ori 3–1 cells (Fig 1B and 1C). Interestingly, we also identified downregulation of E-cadherin level in K1 cells (Fig 1B and 1C). We further measured the level of β-catenin and E-cadherin in membrane fraction of Nthy-ori 3–1 and K1 cells. In membrane fractions, the level of β-catenin and E-cadherin were decreased in K1 cells (Fig 1D and 1E). We then compared the level of β-catenin in nuclear and cytoplasmic fractions from Nthy-ori 3–1 and K1 cells. In both fractions, the level of β-catenin is significantly higher in K1 cells than Nthy-ori 3–1 cells (Fig 1F and 1G). The β-catenin functions as a transcription factor, so the increased level of β-catenin may cause enhanced expression of its target genes, such as c-myc and Cyclin D1. The RT-PCR data confirmed that the transcriptional level of both c-myc and Cyclin D1 was significantly increased in K1 cells (Fig H. In summary, these data suggest that K1 cells have enhanced Wnt signaling.

### K1 cells depend on enhanced Wnt signaling for survival

**Fig 2:**
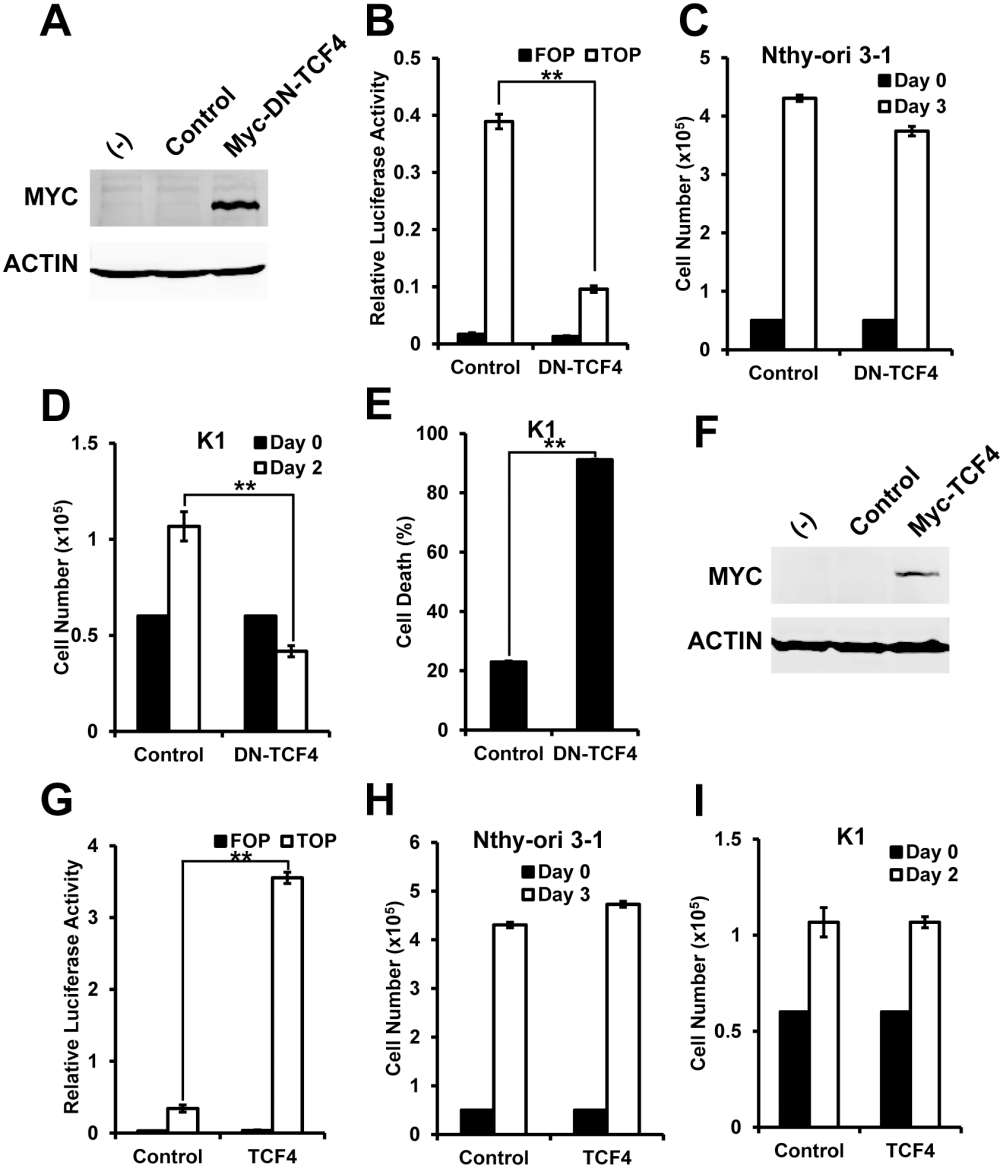
K1 cells depend on Wnt signaling for growth and survival. (A) Immuno blot showed overexpression of DN-TCF4. Actin serves as a loading control; (B) TOP-Flash reporter showed overexpression of DN-TCF4 inhibited the Wnt signaling. FOP-Flash served as a control; (C) and (D) Cell growth was measured in Nthy-ori 3–1 (C) and K1 cells (D) with overexpression of DN-TCF4; (E) Cell death was measured in K1 cells with overexpression of DN-TCF4 by Annexin V and PI assay; (F) Immuno blot showed overexpression of TCF4. Actin serves as a loading control; (G) TOP-Flash reporter showed overexpression of TCF4 enhanced the Wnt signaling. FOP-Flash served as a control; (H) and (I) Cell growth was measured in Nthy-ori 3–1 (H) and K1 cells (I) with overexpression of TCF4. **<0.01.

Considering the enhanced Wnt signaling in K1 cells, we hypothesize that K1 cells may depend on the enhanced Wnt signaling for growth and survival. To test this idea, we used dominant negative (DN)-TCF4 to inhibit Wnt signaling in both normal and cancer cells, and compared the effect on cell growth. The western blot result confirmed the overexpression of DN-TCF4 (Fig 2A). And TOP/FOP-Flash reporter clearly showed that DN-TCF4 inhibited the Wnt signaling in cells (Fig 2B). With the inhibition of Wnt signaling by DN-TCF4, the growth of Nthy-ori 3–1 cells showed minor change (Fig 2C). However, the growth of K1 cells was significantly inhibited (Fig 2D). We next measured cell death by Annexin V and PI cell death assay. We found that Nthy-ori 3–1 cells with DN-TCF4 have no significant cell death (data not shown), and K1 cells with DN-TCF4 showed about 4 folds of increase in cell death (Fig 2E). It suggests that inhibition of Wnt signaling by DN-TCF4 specifically induces cell death in K1 cells. And K1 cells require enhanced Wnt signaling for growth and survival.

We are next to study whether enhancing Wnt signaling by overexpression of TCF4 affects the growth of cancer cells or normal cells. The western blot result showed the overexpression of TCF4 (Fig 2F). And TOP/FOP-Flash reporter confirmed that overexpression of TCF4 significantly enhanced Wnt signaling (Fig 2G). However, no significant change in cell growth was found in both Nthy-ori 3–1 and K1 cells (Fig 2H and 2I).

### Enhanced E2F activity induces cell death in K1 cells

**Fig 3:**
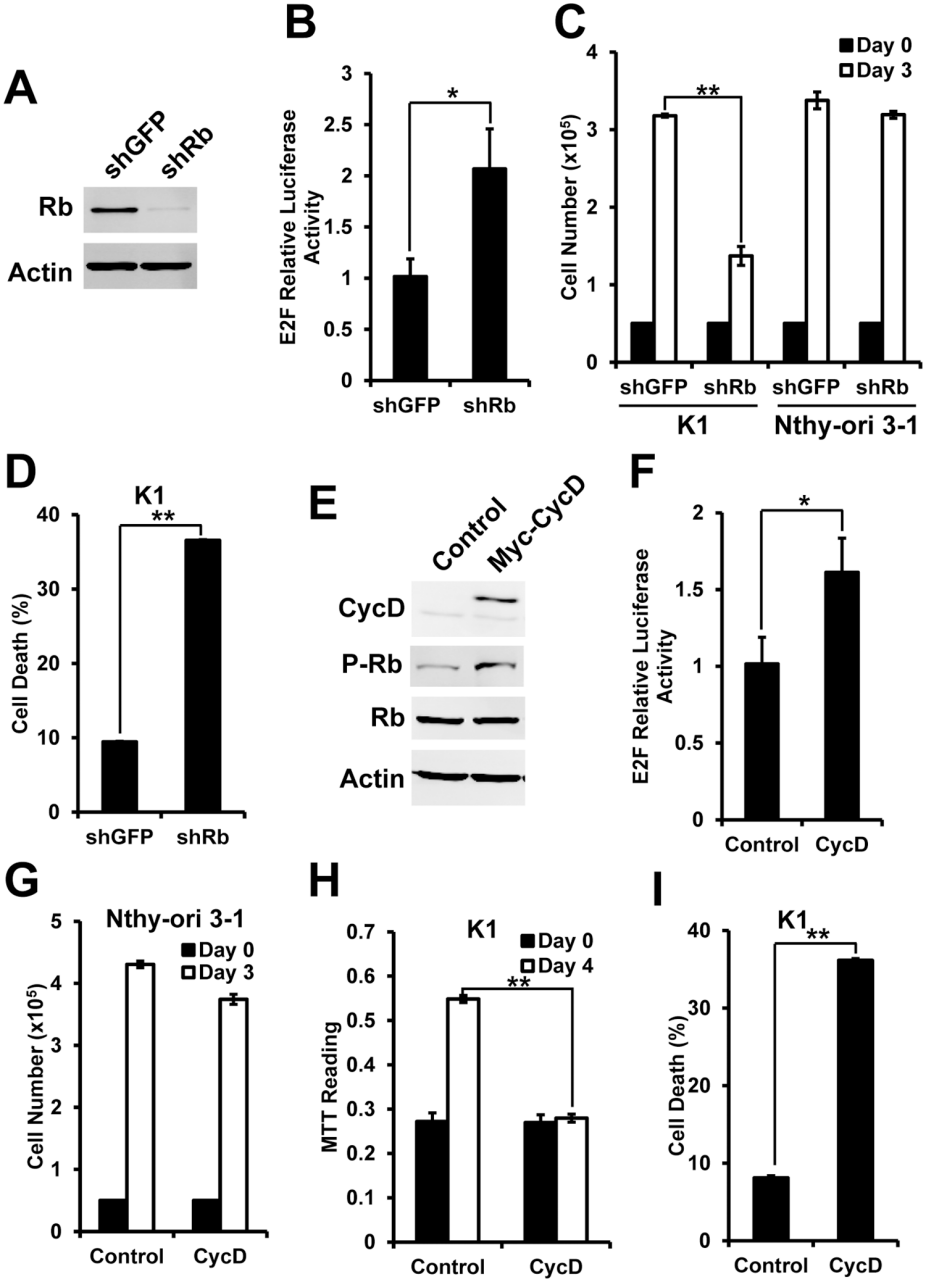
Enhanced E2F activity induced cell death in K1 cells. (A) Immuno blot showed decreased Rb level in cells with knockdown of Rb. Actin serves as a loading control; (B) E2F activity reporter showed knockdown of Rb increased E2F activity; (C) Cell growth was measured in Nthy-ori 3–1 and K1 cells with knockdown of Rb; (D) Cell death was measured in K1 cells with knockdown of Rb by Annexin V and PI assay; (E) Immuno blot showed overexpression of Cyclin D1 and increased level of phosphor-Rb. Rb and Actin serves as loading controls; (F) E2F activity reporter showed overexpression of Cyclin D1 increased E2F activity; (G) and (H) Cell growth was measured in Nthy-ori 3–1 (G) and K1 cells (H) with overexpression of Cyclin D1; (I) Cell death was measured in K1 cells with overexpression of Cyclin D1 by Annexin V and PI assay on Day 4. *<0.05, **<0.01.

Enhanced Wnt signaling was also found in some colorectal cancer cells. Previous studies reported that enhancing E2F activity in these colorectal cancer cells induced cell death effect [7]. So we are interested to test whether the effect is in K1 cells. E2F activity is under the direct negative regulation of Rb. We used shRNA to knockdown endogenous Rb level to enhance E2F activity (Fig 3A). E2F activity luciferase reporter showed that cells with knockdown of Rb have significantly higher E2F activity than control cells (Fig 3B). We next compared cell growth. In Nthy-ori 3–1 cells, no significant change in cell growth was found after knockdown of Rb. However, K1 cells with knockdown of Rb showed significantly decreased cell growth (Fig 3C). The cell death assay further confirmed the enhanced cell death in K1 cells with knockdown of Rb (Fig 3D). It suggests that enhancing E2F activity by knockdown of Rb induced cell death in K1 cells.

As known, E2F activity is also under the regulation of CDK4/6 and Cyclin D1. CDK4/6 and Cyclin D1 phosphorylate Rb and free E2F. Therefore, E2F activity is enhanced without the negative regulation of Rb. Next, we like to further confirm whether indirectly enhancing E2F activity by overexpression of Cyclin D1 also causes cell death. We first confirmed that overexpression of Cyclin D1 by western blot. And we also found that overexpression of Cyclin D1 increased the level of phosphor-Rb in K1 cells (Fig 3E). E2F luciferase reporter also showed increased E2F activity in cells with overexpression of Cyclin D1 (Fig 3F). We next found that overexpression of Cyclin D1 in Nthy-ori 3–1 cells caused no effect in cell growth and cell death (Fig 3G). However, overexpression of Cyclin D1 caused decreased cell growth and enhanced cell death in K1 cells (Fig 3H and 3I). It suggests that indirectly enhancing E2F activity by overexpression of Cyclin D1 also specifically induced cell death in K1 cells.

### Oxidative stress mediates cell death caused by knockdown of Rb in K1 cells

**Fig 4:**
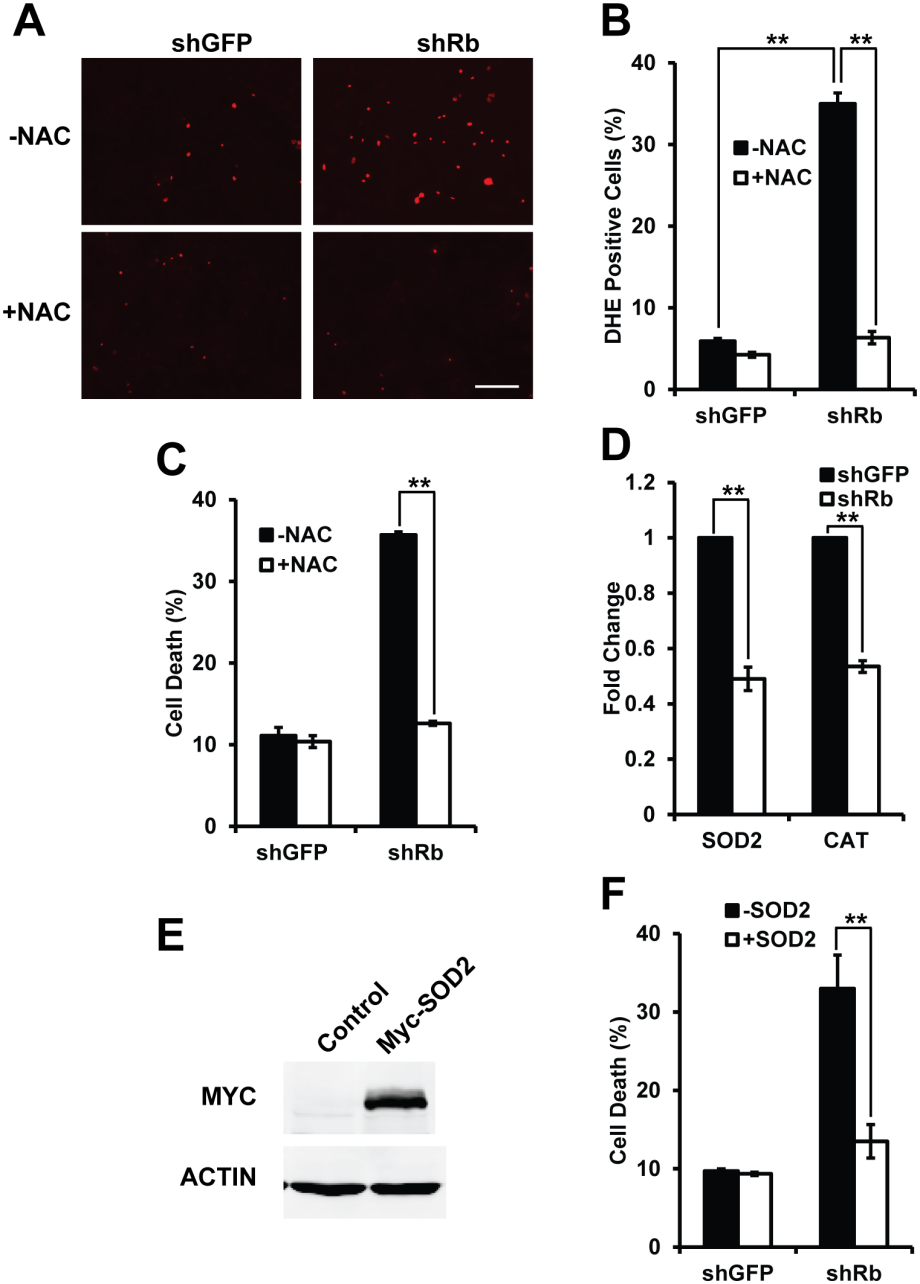
Oxidative stress induced by knockdown of Rb caused the cell death in K1 cells. (A) Cell stained with Dihydroergotamine (DHE) showing increased DHE fluorescence in K1 cells with knockdown of Rb, and adding N-acetyl cysteine (NAC, 5mM) decreased the DHE signal. Scale bar, 100 μm; (B) Quantification of the percentage of DHE positive cells in (A); (C) Cell death measured by Annexin V and PI assay showing adding NAC rescued the cell death in K1 cells with knockdown of Rb. (D) mRNA expression of SOD2 and Catalase (CAT) relative to the level of GAPDH in K1 cells with knockdown of Rb. (E) Immuno blot showed overexpression of SOD2. Actin serves as a loading control; (F) Cell death measured by Annexin V and PI assay showing overexpression of SOD2 rescued the cell death in K1 cells with knockdown of Rb. **<0.01.

Previous studies showed that the induced cell death may be related to the enhanced oxidative stress [7]. So we hypothesize that knockdown of Rb in K1 cells may induce oxidative stress. To test it, we used Dihydroergotamine (DHE), a dye that detects superoxide, to determine whether knockdown of Rb induces oxidative stress in K1 cells [9]. Comparing to control cells, we found significantly more DHE positive cells in the cells with knockdown of Rb (Fig 4A and 4B). It suggests that knockdown of Rb in K1 cells induces oxidative stress. And the cell death by knockdown of Rb might be due to enhanced oxidative stress. To test this idea, the antioxidant N-acetyl cysteine was used to reduce oxidative stress. Addition of NAC significantly decreased the oxidative stress in the cells with knockdown of Rb (Fig 4A and 4B). Furthermore, addition of NAC significantly rescued the cell death in the cells with knockdown of Rb (Fig 4C).

To further study how enhanced E2F activities in K1 cells induced oxidative stress, we first measured the gene expression of ROS scavenger enzymes in cells. SOD2 and catalase are two well-known ROS scavenger enzymes. We found that the expressions of Sod2 and catalase were significantly inhibited in cells with knockdown of Rb (Fig 4D). Overexpression of SOD2 in cells with knockdown of Rb significantly rescued the cells from cell death (Fig 4E and 4F). Summarily, these data strongly support that knockdown of Rb induces oxidative stress, and further causes cell death in K1 cells.

## Discussion

In this study, we demonstrated the important roles of Wnt signaling in the K1 cells. Consistent with its important roles, Wnt signaling is enhanced in K1 cells in comparison to normal thyroid cells. More importantly, the enhanced Wnt signaling is required for the growth and survival of K1 cells. Previous studies indicated that enhanced Wnt signaling is a later event in the development of thyroid tumor, because the most frequent genetic mutations in Wnt signaling genes APC, Axin or β-catenin were found in later stage poorly differentiated and ATC, instead of PTC [10,11]. However, this conclusion was challenged by recent studies done in TPC1 cells, which are PTC cells. The studies found that the RET/PTC chromosomal mutation in TPC1 cells result in the fusion of the tyrosine kinase domain of the RET receptor with the NH2 terminus of heterologous proteins, thereby generating the RET/PTC oncoproteins. The RET/PTC oncoproteins stimulates the β-catenin, and therefore enhances Wnt signaling [4,6]. In our study, without RET/PTC chromosomal mutation, K1 cells still have enhanced Wnt signaling, and rely on it for growth and survival. These findings present a novel cell example to support the important function of Wnt signaling in earlier stage of thyroid tumor.

The detail mechanism of activation of Wnt signaling in K1 cells is still largely unknown. However, our studies support that the enhanced Wnt signaling in K1 cells may be related to the decreased level of E-cadherin. In K1 cells, we identified decreased level of E-cadherin and β-catenin in membrane fraction along with increased level of β-catenin in both cytoplasmic and nuclear fractions (Fig 1B–1G). Previous studies showed that E-Cadherin is a transmembrane protein that mediates cell-cell adhesion in a Ca^2+^-dependent manner. It interacts through its cytoplasmic domain with β-catenin and the actin cytoskeleton, controlling cell migration and cell polarity [12]. E-cadherin keeps β-catenin bound to the cell membrane, so the increased presence of cytoplasmic and nuclear β-catenin in K1 cells could be merely a consequence of the loss of E-cadherin expression. Previous studies support that the loss of E-cadherin might be due to the activation mutation BRAF (V600E) in K1 cells [13,14]. Studies showed that the activation mutation BRAF (V600E) downregulates the level of E-cadherin, and implicated in the induction of the epithelial mesenchymal transition in follicular, papillary, and anaplastic thyroid tumor cells [15]. However, these detail mechanism need to be verified by further studies.

Remarkably, we revealed that increasing E2F activity by either knockdown of Rb or overexpression of Cyclin D1 leads to cell death in K1 cells. We further identified that the cause of the cell death is the enhanced oxidative stress. Similar cell phenotypes were identified in colorectal cancer cells, which often have enhanced Wnt signaling. The studies found that knockdown of Rb enhanced cell stress and induced cell death [7]. The mechanism by which cellular stresses induced is complex and is not entirely clear. In our study, we found that enhanced E2F activities specifically regulate gene expression of ROS scavenger enzymes. Rb/E2F pathway has significant impact on metabolic pathways, and plays an important role in the regulation of redox homeostasis [16,17,18,19]. Previous studies suggested that there are different ways that Rb/E2F can regulate ROS through SOD2 proteins. Tanaka *et al*. showed that E2F1 induced ROS accumulation in cells, and suppressed ROS-induced SOD2 expression by inhibiting NF-kB activity [20]. Imai *et al*. showed that Rb cooperated with AKT signaling to regulate the expression of SOD2 through FoxM1 [21]. Some studies found that the oxidative stress might result from other stresses, such as metabolic stress and ER stress [17], which are induced by knockdown of Rb. Further studies are required to reveal the details.

In conclusion, we found the important function of Wnt signaling in the growth and survival of K1 cells. It indicates the key roles of Wnt signaling in the early development of thyroid tumor. We also identified the cell death effect in K1 cells by enhancing E2F activity. It provides an alternative way to limit thyroid tumor.
